# Long-Term Survey Is Necessary to Reveal Various Shifts of Microbial Composition in Corals

**DOI:** 10.3389/fmicb.2017.01094

**Published:** 2017-06-13

**Authors:** Shan-Hua Yang, Ching-Hung Tseng, Chang-Rung Huang, Chung-Pin Chen, Kshitij Tandon, Sonny T. M. Lee, Pei-Wen Chiang, Jia-Ho Shiu, Chaolun A. Chen, Sen-Lin Tang

**Affiliations:** ^1^Biodiversity Research Center, Academia SinicaTaipei, Taiwan; ^2^Germark Biotechnology Co., Ltd.Taichung, Taiwan; ^3^Yourgene BioscienceNew Taipei City, Taiwan; ^4^Bioinformatics Program, Institute of Information Science, Taiwan International Graduate Program, Academia SinicaTaipei, Taiwan; ^5^Institute of Bioinformatics and Structural Biology, National Tsing Hua UniversityHsinchu, Taiwan; ^6^Section of Gastroenterology, Hepatology and Nutrition, Department of Medicine, University of Chicago Medicine, ChicagoIL, United States; ^7^Molecular and Biological Agricultural Sciences Program, Taiwan International Graduate Program, Academia SinicaTaipei, Taiwan; ^8^Graduate Institute of Biotechnology, National Chung Hsing UniversityTaichung, Taiwan

**Keywords:** long-term survey, microbial composition, corals, *Stylophora pistillata*, shifts

## Abstract

The coral holobiont is the assemblage of coral host and its microbial symbionts, which functions as a unit and is responsive to host species and environmental factors. Although monitoring surveys have been done to determine bacteria associated with coral, none have persisted for >1 year. Therefore, potential variations in minor or dominant community members that occur over extended intervals have not been characterized. In this study, 16S rRNA gene amplicon pyrosequencing was used to investigate the relationship between bacterial communities in healthy *Stylophora pistillata* in tropical and subtropical Taiwan over 2 years, apparently one of the longest surveys of coral-associated microbes. Dominant bacterial genera in *S. pistillata* had disparate changes in different geographical setups, whereas the constitution of minor bacteria fluctuated in abundance over time. We concluded that dominant bacteria (*Acinetobacter, Propionibacterium*, and *Pseudomonas*) were stable in composition, regardless of seasonal and geographical variations, whereas *Endozoicomonas* had a geographical preference. In addition, by combining current data with previous studies, we concluded that a minor bacteria symbiont, *Ralstonia*, was a keystone species in coral. Finally, we concluded that long-term surveys for coral microbial communities were necessary to detect compositional shifts, especially for minor bacterial members in corals.

## Introduction

Corals harbor a variety of microorganisms, including algae, fungi, bacteria, archaea, and viruses (Knowlton and Rohwer, 2003; Wegley et al., 2007; Marhaver et al., 2008). Coral-associated microbes, which live in coral mucus, tissue, and skeleton, have various interactions with their host and maintain coral holobiont function, including antimicrobial defense and nutrient acquisition (Bourne et al., 2016). With advances in high-throughput sequencing technologies, there is growing research interest in microbial ecology in rare biospheres (Lynch and Neufeld, 2015), characterizing genetic and functional diversity supporting global ecosystem health (Yachi and Loreau, 1999; Elshahed et al., 2008).

Composition of coral-associated microorganisms is affected by coral species (Littman et al., 2009; Reis et al., 2009; Kvennefors et al., 2010; Lee et al., 2012; Lema et al., 2012; McKew et al., 2012; Morrow et al., 2012; Carlos et al., 2013), environmental factors (Hong et al., 2009; Ceh et al., 2011; Chen et al., 2011), geographical differences (Hong et al., 2009; Littman et al., 2009; Kvennefors et al., 2010; Lee et al., 2012; Morrow et al., 2012; Lema et al., 2014), and human activities (Klaus et al., 2007). According to previous studies, some microbes had host specificity, regardless of geographical difference (Rohwer et al., 2001, 2002), whereas others had regional specificity rather than host types (Littman et al., 2009).

Various coral-associated microbial species reportedly shared similar functional roles in coral ecology (Li et al., 2013). However, bacterial succession in corals has not been well characterized, as the longest monitoring survey lasted only 1 year (Rohwer et al., 2002; Hong et al., 2009; Ceh et al., 2011; Carlos et al., 2013; Lema et al., 2014; Hernandez-Agreda et al., 2016). Furthermore, although some microbes were consistently detected in coral (Rohwer et al., 2002; Carlos et al., 2013), it is still unknown whether these common microbes change their abundance over longer intervals. A 2-years survey characterizing dynamics of *Symbiodinium* rare biosphere provided evidence for symbiont switching in reef-building corals after environmental changes (Boulotte et al., 2016), but dynamics of the bacterial rare biosphere in coral, and bacterial shifting/switching within corals, are not established.

The objective was to determine whether a long-term survey was necessary to detect compositional dynamics of coral-associated bacteria in various locations. Based on previous short-term studies, we hypothesized that coral-associated bacteria require varying time scales to manifest detectable variations in different environments. Cumulative stressors, e.g., nutrient availability and elevated sea surface temperature, have been postulated to change coral reef ecosystems; therefore, coral-associated microbes might either attenuate or exacerbate effects from stressors through their roles in coral holobiont fitness (Bourne et al., 2008, 2016). Since cumulative stressors vary from place to place, duration of detectable changes in coral-associated microbes is also likely to vary, which should be apparent in a long-term survey (Mouchka et al., 2010; Ceh et al., 2011; Chen et al., 2011; Lee et al., 2012).

To determine dynamics of coral-associated microbial communities among locations and to study temporal variations of dominant and minor bacterial groups, 16S rRNA gene amplicon pyrosequencing was used in a 2-years observational study investigating bacterial communities in coral *Stylophora pistillata*, which is widely distributed in Taiwan and around the globe. That Taiwan lies across the Tropic of Cancer, *S. pistillata* in tropical (Kenting and Lyudao) and subtropical (Yehliu) Taiwan were collected to provide a comparative basis for latitude influence on bacterial composition in corals. This 2-years survey was to assess coral-associated microbial differences among various climate zones, and determine importance of long-term observation to detect bacterial dynamics in coral hosts.

## Materials and Methods

### Coral Samples

**FIGURE 1:**
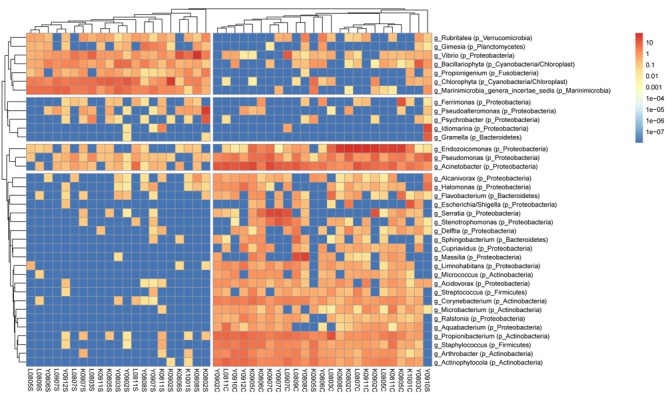
Clustering analysis of bacterial genera in *S. pistillata* and seawater. A total of 37 genera remained in the analysis after excluding genera of unknown taxonomy or <0.2% relative abundance. Trees were cut at 2 and 4 groups in columns and rows, respectively. Within each sample, the color scale indicates relative abundance (percentage). Genus names are prefixed with “g_,” and phylum names with “p_” in parenthesis. Columns are clustered by Bray–Curtis distance and rows by Euclidean distance of their abundance profile.

Coral and seawater samples were collected from three locations, Kenting, Yehliu, and Lyudao, in Taiwan (**Figure 1**) from February 2008 to January 2010. In each location and at each sampling time, three colonies (replicates) of *S. pistillata* at depths of 3–7 m were selected and tagged for sampling. For each colony, a coral fragment approximately 2 cm long and 2 cm in diameter was collected. Samples were washed twice with filtered seawater and stored at 4°C until coral tissue was processed. In addition, 1 L of seawater surrounding the coral colony was simultaneously collected to analyze the microbial community in reef water. A total of 88 *S. pistillata* and seawater samples were collected (Supplementary Table S1). Environmental data (precipitation, daylight duration and air and water temperatures) were obtained from the Central Weather Bureau in Taiwan (Supplementary Table S1).

Coral tissues were sprayed (using an airbrush) with 2–5 mL 10× TE buffer (10 mM Tris–HCl, pH 8.5, 1 mM EDTA, pH 8.0) and collected in a sterilized bag. Coral tissues were transferred to a 15 mL sterilized tube and stored at -20°C pending DNA extraction.

### DNA Extraction, Primers, PCR, and Sequencing

Coral tissue was recovered for genomic DNA extraction by centrifugation (10,000 × *g*, 15 min). After discarding the supernatant, tissue pellets were put into 600 μL of 10× TE buffer and liquid nitrogen, homogenized (mortar and pestle), and the resulting solution transferred to a clean tube. Seawater samples were filtered through membrane filters (0.2 μm pore size; Adventec, Tokyo, Japan) to collect microbial particulate. Total genomic DNA of coral tissue and seawater were extracted as outlined in the UltraClean Soil DNA Kit (MioBio, Solana Beach, CA, United States).

The PCR was performed using two bacterial universal primers: 27F (5′-AGAGTTTGATCMTGGCTCAG-3′) and 341R (5′-CTGCTGCCTCCCGTAGG-3′), which were designed for the V1–V2 hypervariable region of the bacterial 16S rRNA gene (Hamp et al., 2009). The reaction mixture contained 1 μL of 5 U TaKaRa *Ex Taq* HS (Takara Bio, Otsu, Japan), 5 μL of 10×*Ex Taq* buffer, 4 μL of 2.5 mM deoxynucleotide triphosphate mixture, 1 μL of each primer (10 μM), and 1–5 μL (10–20 ng) template DNA in a volume of 50 μL. The temperature program for 30 PCR cycles was the initial step of 94°C for 3 min, 94°C for 20 s, 52°C for 20 s, 72°C for 20 s, and 72°C for 2 min (final extension after the last cycle).

The PCR amplicons of the bacterial 16S rRNA gene V1–V2 region were verified by DNA agarose gel electrophoresis with 1.5% agarose gel and 10× TE buffer. The expected DNA band (∼320 bp) was cut from the gel and DNA was recovered with a QIAEX II Agarose Gel Extraction kit (QIAGEN, Hilden Germany) and quality verified with a Scandrop spectrophotometer (Thermo Scientific, Vantaa, Finland).

A DNA tagging PCR was used to tag each PCR product of the bacterial 16S rRNA gene V1–V2 region (Chen et al., 2011). According to Chen et al. (2011), the tag primer was designed with four overhanging nucleotides; this arrangement ensured 256 distinct tags, at the 5′ end of the 27F and 341R primers for bacterial DNA. The tagging PCR conditions consisted of an initial step of 94°C for 3 min, 5 cycles of 94°C for 20 s, 60°C for 15 s, 72°C for 20 s, and a final step at 72°C for 2 min.

Pooled 40 ng lots of each tagged bacterial 16S rRNA gene V1–V2 region DNA samples (88 samples in total) were used for massively parallel pyrosequencing using a Roche 454 Genome Sequencer FLX System (Mission Biotech, Taipei, Taiwan). Raw sequencing reads were sorted according to unique tags using an in-house script, and deposited in NCBI Sequence Read Archive database, under accession number SRP098878, after the primer was removed. Reads from replicate colonies, sampled at the same time and location, were combined into one sample.

### Data Analysis

On a per-sample basis, bacterial reads were quality-filtered using Mothur v1.36.1 (Schloss et al., 2009) to retain reads of lengths from 290 to 340 base pairs (bp), and an average quality score >27. Reads containing any ambiguous base or homopolymer >8 bp were excluded. Chimeric reads were detected and removed using USEARCH v8.1.1861 (Edgar, 2010) with reference mode (3% minimum divergence). Quality-filtered and non-chimeric reads were analyzed using UPARSE pipeline (Edgar, 2013) to generate OTUs per sample (97% identity level), which were classified with RDP classifier (v2.12) (Wang et al., 2007) with a pseudo-bootstrap threshold of 80%.

The rarefied diversity measures (e.g., Shannon, Simpson, Chao 1, ACE, and Good’s coverage) were estimated using Mothur with 1000 iterations. The sample size (*N*) varied from 567 to 29819; therefore, 567 was chosen for rarefaction analysis.

### Cross-Sample OTU Identification of *Acinetobacter* and *Endozoicomonas*

Bacterial OTU representative sequences of all samples affiliated with a given taxonomy were collectively clustered using USEARCH, with options –cluster_smallmem and –id 0.97. Abundance of each centroid (or cross-sample OTU) was represented by average relative abundance of its cluster members. Phylogenetic analysis was conducted in MEGA7 (Kumar et al., 2016), with maximum-likelihood trees generated with 1000 bootstraps for aligned representative sequences.

### Resident Types of Bacterial Lineages

The present study defined six resident types of bacteria in *S. pistillata* and seawater, based on relative abundance and detection frequency. Type I (prevalent abundant group), type II (moderately prevalent abundant group), and type III (non-prevalent abundant group) each had ≥1% average relative abundance and were detected in ≥80, 80–60, and <60% samples, respectively. Type IV (prevalent minor group), type V (moderately prevalent minor group), and type VI (non-prevalent minor group) had <1% average relative abundance and were detected in ≥80, 80–60, and <60% samples, respectively. Coral and seawater samples were separately considered (in terms of detection frequency).

### Clustering Analysis of Bacterial Taxa

Relative abundance of each classified bacterial genera in individual samples was incorporated into a matrix to estimate the Bray–Curtis distance. Results were presented by non-metric multidimensional scaling (nMDS) analysis using Primer 6 (PRIMER-E, Lutton, Lvybridge, United Kingdom; Clarke and Warwick, 2001) to determine relationships of bacterial communities among times and locations. Analysis of similarity (ANOSIM) via Primer 6 was used to test bacterial diversity indices and compositions in spatiotemporal variations.

For analysis of abundant and minor bacterial members, abundant groups (type I–III) and minor groups (type IV–VI) were separately incorporated into two matrixes. These matrices were used to perform ANOSIM in a Primer 6 based Bray–Curtis distance derived from relative abundance transformed by three methods, including no transformation, square root transformation, and binary transformation (i.e., presence and absence analysis).

Bacterial compositions between sampling times in each location were compared with Pearson correlations using R package Hmisc^1^. In addition, Pearson correlations were used to compare fluctuations of *Endozoicomonas* and *Acinetobacter*.

Hierarchical clustering analysis was performed using the average linkage via R package pheatmap (Kolde, 2015) and visualized on a heat map. The distance matrix used for clustering was calculated using the R package vegan (Dixon, 2003) based on a base-10 logarithmic transformation of relative abundance (in percentage), plus a pseudo-count of 1 × 10^-8^ (Costea et al., 2014). Genera of unknown taxonomy or <0.2% relative abundance were excluded.

## Results

### Diversity of Microbial Community

Seawater sample K1001S yielded the most operational taxonomic units (OTUs), 406.15 (in rarefaction), whereas coral sample K0808C had the fewest (58.68 OTUs; Supplementary Table S2). According to ANOSIM, bacterial diversity indices in corals and seawater samples differed (richness: *R* = 0.634, *p* = 0.001; Shannon: *R* = 0.298, *p* = 0.001) and bacterial community in seawater samples had greater richness and Shannon diversity than those in corals (Supplementary Table S2).

### Composition of Bacterial Community

Overall, bacterial compositions in *S. pistillata* from Kenting, Yehliu, and Lyudao were similar at a class level, similar to seawater samples (Supplementary Figure S1). However, bacterial communities in *S. pistillata* and seawater differed from each other. Dominant bacterial classes in seawater were Flavobacteria, unclassified Marinimicrobia, Gammaproteobacteria, and Alphaproteobacteria. Among these, most dominant genera were unclassified, such as unclassified Marinimicrobia, unclassified Gammaproteobacteria, and unclassified Flavobacteriaceae. In addition, the genus *Vibrio*, which belongs to Gammaproteobacteria, was higher than those in corals.

In *S. pistillata*, Actinobacteria, Betaproteobacteria, and Gammaproteobacteria were dominant, and their relative abundances higher than those in seawater. In Gammaproteobacteria, dominant genera were *Endozoicomonas, Acinetobacter, Pseudomonas*, and *Serratia* (Supplementary Figure S1), among which *Endozoicomonas* was particularly more dominant in Kenting and Lyudao compared to Yehliu. *Propionibacterium* and *Actinophytocola* were the two dominant Actinobacteria genera, and *Massilia* Betaproteobacteria in coral.

Bacterial community structure in *S. pistillata* and seawater at various sampling times is shown (**Figure 1**). Some genera, such as *Ralstonia, Cupriavidus*, and *Aquabacterium*, were only detected in coral samples, whereas *Pseudoalteromonas* was common only in seawater, and *Gramella* was exclusively retrieved from seawater samples. In addition, among coral samples, although some bacterial taxa were prevalent at every collection, their relative abundance varied from time to time (**Figure 1**). For example, *Endozoicimonas* and *Acinetobacter* fluctuated in abundance throughout this survey.

### Variation of Bacterial Composition in Different Time

**FIGURE 2:**
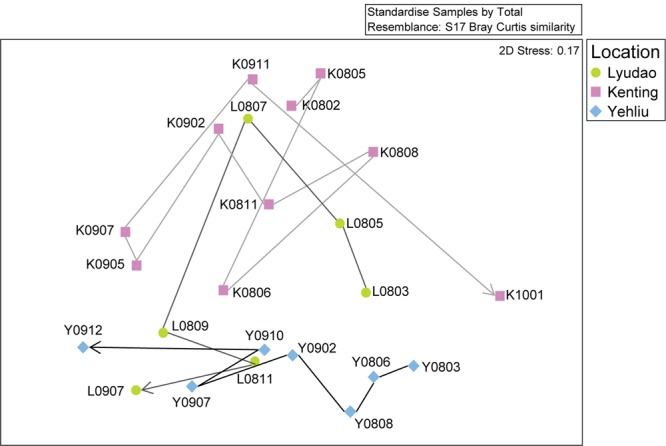
Non-metric multidimensional scaling (nMDS) analysis of the changing *S. pistillata*-associated bacterial communities in three locations at various times (2008–2010). Symbols indicate samples from different locations; circles are for Lyudao, squares for Kenting, and diamonds for Yehliu. The lines and arrow indicate the change of bacterial communities from the first to the last sampling time and colors of lines distinguish locations.

There were differences among sampling years in bacterial community structure (ANOSIM: *R* = 0.301, *p* = 0.002; Supplementary Figure S2). Among all sites, based on nMDS, the bacterial community changed along the sampling time (**Figure 2**). Within each location, bacterial composition also differed among sampling years or times (Supplementary Figure S3), especially in samples from Kenting (ANOSIM: *R* = 0.370, *p* = 0.028). In Lyudao and Yehliu, bacterial compositions were clustered according to sampling years (difference not significant), but the Pearson correlation value between each sampling time in Lyudao and Yehliu decreased significantly (in Lyudao, from 1.00 to 0.24; in Yehliu, from 1.00 to 0.16) with increasing time span (Supplementary Figure S4), which indicated changes in bacterial composition during sampling times. In addition, there was no difference between seasons (ANOSIM: *R* = -0.072, *p* = 0.791).

**FIGURE 3:**
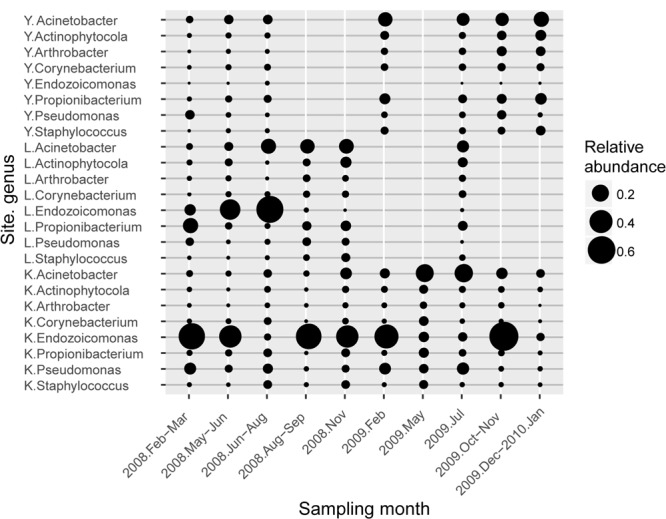
Average abundance profile of resident type-I genera associated with *S. pistillata*. Circle size represents the average relative abundance of each genus. Each genus name was prefixed with a one-letter abbreviation for sampling sites (K is for Kenting, L for Lyudao, and Y for Yehliu).

To determine differential effects of short- versus long-term observations, consecutive patterns of correlations between bacterial communities were analyzed (Supplementary Figure S5). Correlations fluctuated over five consecutive samplings (Supplementary Figures S5A–F). However, a longer time span revealed a more holistic view of variation in bacterial composition (Supplementary Figure S5G), not apparent in short-term observations. The longer time span also revealed that changes followed a pattern. For example, two peaks (times 4–6 and time 9) had different duration, indicating different resilience status (Supplementary Figure S5G). Both abundant and minor (<1% relative abundance) genera had a significant differences between sampling years, and only minor genera did so by considering presence/absence data (binary transformation; Supplementary Table S3). In Yehliu, these abundant genera (except *Endozoicomonas*) retained similar abundance and moderately increased in 2009, although they had more variable fluctuations in abundance in Lyudao and Kenting, especially *Endozoicomonas* and *Acinetobacter* (**Figure 3**). In Lyudao, *Acinetobacter* increased during July to November in 2008, and *Endozoicomonas* increased during May and July in 2008, but decreased thereafter. In Kenting, abundance of *Acinetobacter* was low during 2008, increased during May and July in 2009, and decreased from November 2009 to January 2010. However, *Endozoicomonas* bloomed in most sampling times during 2008 and decreased during May and July in 2009, followed by an increase in November 2009. Intriguingly, both in Lyudao and Kenting, the times of *Endozoicomonas* and *Acinetobacter* blooms were staggered (**Figure 3**). Furthermore, in Kenting, there was a negative correlation (Pearson correlation: *R* = -0.899, *p* = 0.038) between *Endozoicomonas* and *Acinetobacter* abundance fluctuations from November 2008 to November 2009.

### Variation of Bacterial Composition among Locations

Based on nMDS, there was distinct clustering of coral-associated bacterial composition in Yehliu, Lyudao, and Kenting (ANOSIM: *R* = 0.224, *p* = 0.014; **Figure 2**). Kenting and Yehliu had differences not only in bacterial composition (ANOSIM: *R* = 0.437, *p* = 0.004) but also in community richness (ANOSIM: *R* = 0.218, *p* = 0.045). There was a compositional difference among sampling sites in dominant genera (ANOSIM: *R* = 0.240, *p* = 0.008), but not in minor genera (Supplementary Table S3).

**FIGURE 4:**
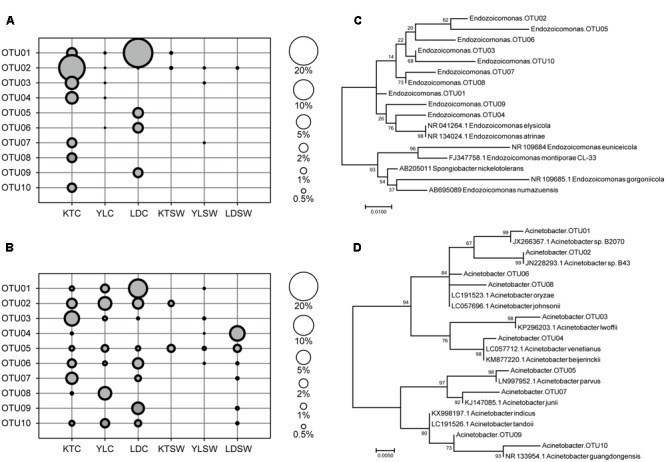
Top-10 abundant OTU profiles and molecular phylogenetic analysis of *Endozoicomonas* and *Acinetobacter* in *S. pistillata* and seawater. The abundance profile of top-10 OTUs of **(A)** *Endozoicomonas* and **(B)** *Acinetobacter* were organized in a descendent order according to their average relative abundance (scaled by circle size). Maximum-likelihood trees (with 1000 bootstraps) among OTUs of **(C)** *Endozoicomonas* and **(D)** *Acinetobacter* were also displayed. KT, Kenting; YL, Yehlui; LD, Lyudao; C, colony; SW, seawater.

*Endozoicomonas* and *Acinetobacter* were the two frequently detected genera of high abundance (**Figure 3**), of which both had disparate inhabiting preferences on *S. pistillata* in different locations (**Figure 4**). *Endozoicomonas* were mostly present in corals from Lyudao and Kenting, and composed of distinct genotypes in each locations (i.e., OTU02, 03, 04, 07, 08, and 10 in Kenting; OTU05, 06, and 09 in Lyudao). These *Endozoicomonas* OTUs were merely detected in both Kenting and Lyudao, indicating that geographical setups determined their inhabiting specificity (**Figure 4A**). Based on phylogenetic analysis, these OTUs were highly associated with *E. elysicola* and *E. atrinae* (**Figure 4C**). In contrast to exclusive detection of *Endozoicomonas* in coral, *Acinetobacter* were present in both *S. pistillata* and seawater (**Figure 4B**). Furthermore, most *Acinetobacter* OTUs were shared by three sampling sites, with less geographical preference than *Endozoicomonas* (**Figure 4B**). These *Acinetobacter* OTUs were also more diverse compared to *Endozoicomonas* OTUs in terms of phylogenetic affiliation (**Figure 4D**).

### The Resident Types of Coral-Associated and Seawater-Associated Bacteria

According to relative abundance and detection frequency, bacteria in *S. pistillata* and seawater were categorized into six resident types (Section Resident Types of Bacterial Lineages in Materials and Methods and **Table 1**). In *S. pistillata*, type I group (i.e., with >1% relative abundance and detected in ≥80% samples) had eight genera, including *Acinetobacter, Endozoicomonas, Propionibacterium, Corynebacterium, Staphylococcus, Arthrobacter, Actinophytocola*, and *Pseudomonas*. However, there were only *Vibrio* and unclassified *Marinimicrobia* as type I in seawater. Among these abundant genera (resident type I–III), coral and seawater shared no genus.

**Table 1:** Resident types of bacterial genera associated with *S. pistillata* and seawater.

Category	Definition	In *Stylophora pistillata*	In seawater
Type I, prevalent abundant group	≥1% average relative abundance; present in ≥80% samples	*Acinetobacter*^∗^, *Endozoicomonas, Propionibacterium, Corynebacterium, Staphylococcus, Arthrobacter, Actinophytocola, Pseudomonas*^∗^	*Vibrio*^∗^, unclassified Marinimicrobia
Type II, moderately prevalent abundant group	≥1% average relative abundance; present in 60–80% samples	*Serratia, Stenotrophomonas*	*Pseudoalteromonas*
Type III, non-prevalent abundant group	≥1% average relative abundance; present in <60% samples	*Massilia*	-
Type IV, prevalent minor group	<1% average relative abundance; present in ≥80% samples	*Ralstonia, Brevibacterium, Microbacterium, Acidovorax, Limnohabitans, Streptococcus, Aquabacterium, Micrococcus*	*Polaribacter, Propionigenium, Arcobacter, Pseudomonas^∗^, Photobacterium, Rubritalea*
Type V, moderately prevalent minor group	<1% average relative abundance; present in 60–80% samples	*Polynucleobacter, Enhydrobacter, Flavobacterium, Cupriavidus, Comamonas, Alcanivorax, Halomonas, Deinococcus, Burkholderia, Delftia, Vibrio*^∗^, *Chryseobacterium, Anaerococcus*	*Ilumatobacter, Tenacibaculum, Aureispira, Cetobacterium, Phycisphaera, Gimesia, Candidatus* Pelagibacter, *Psychrosphaera, Halioglobus, Acinetobacter*^∗^
Type VI, non-prevalent minor group	<1% average relative abundance; present in <60% samples	318 genera	339 genera

*^∗^Genera detected in both coral and seawater.*

## Discussion

The present study demonstrated that long-term observation of coral-associated microbial communities provided a baseline to elucidate coral–microbe and microbe–microbe interactions. Abundant and minor bacteria in *S. pistillata* had different inclinations to geographical and temporal changes. Based on relative abundance and detection frequency, prevalent and non-prevalent *S. pistillata*-associated bacterial genera were allotted into six putative resident types.

### Long-Term Survey Revealed Various Shifts in Coral-Associated Bacterial Community

Shifts in bacterial communities in healthy coral tissues were only apparent with a sufficient duration of survey (∼2 years). Although changes in bacterial composition in Lyudao and Yehliu were not confirmed (samples were lost due to typhoons and human activities), based on the correlation between sampling times, community resilience may be present over a longer sampling interval. In the present study, the long and complete observation in Kenting successfully captured compositional resilience that was not apparent in short-term surveys. A small group of bacteria (that comprised the core microbiome) were ubiquitously associated with coral, regardless of abiotic environmental parameters (Hernandez-Agreda et al., 2016). However, based on our results, prolonged observation enabled characterizing their succession in coral (Lynch and Neufeld, 2015). In this study, changes in prevalent abundant genera *Acinetobacter* and *Endozoicomonas* had disparate fluctuation patterns among locations, with abundances constant in Yehliu but variable in Kenting in 2009. Negative abundance correlations between these two genera were not detected before November 2008 but afterward until November 2009; therefore, this successional pattern was far from conclusive. Apart from abundant ones, minor bacterial genera in *S. pistillata* also changed in abundance throughout the study, which emphasized the need for longer surveys of coral-associated bacteria. Although Pantos et al. (2003) proposed some explanations for observed fluctuations in coral-associated bacterial communities, their hypotheses were only based on short-term observations. It has been suggested that coral-associated microbes can attenuate or intensify cumulative stressors that may disrupt coral reef ecosystems (Bourne et al., 2016). Therefore, we believe that these stressors, which varied in times and degrees from place to place, also contributed to spatial and temporal dynamics in coral-associated bacterial composition and abundance.

### Abundant Bacterial Genera Showed Differential Geographical Inclinations

Different coral reproduction strategies (i.e., broadcast spawning and brooding) affect symbiotic bacterial composition differently, and *S. pistillata*, a brooder coral, harbored distinguishable bacterial communities strongly clustered by geographical location (Neave et al., 2017b), implying, in *S. pistillata*, vertical transfer of microbes from parents to offspring, thereby restricting the microbial structure and development and leading to the observed geographical grouping. As some bacteria have inhabiting specificity to coral species (Hong et al., 2009; Reis et al., 2009), discrepant microbial compositions among corals reported by different studies were plausible.

Among abundant genera, *Acinetobacter, Propionibacterium*, and *Pseudomonas* were more constantly detected in *S*. *pistillata* regardless of seasonal and geographical differences, whereas *Endozoicomonas* had geographical variations. *Endozoicomonas* spp. have diverse roles in different hosts as being symbiotic (La Rivière et al., 2013) and parasitic (Mendoza et al., 2013). The documented high proportion of repeats and insertion sequences in *E. montiporae* (Ding et al., 2016) and pathogenic strain *Candidatus* Endozoicomonas cretensis (Katharios et al., 2015) corresponded to their high genome plasticity for wide adaptation to various hosts (reviewed by Neave et al., 2017a). Since *Endozoicomonas* was abundant in Lyudao and Kenting but not in Yehliu, their adaptation appeared more favorable for tropical versus subtropical oceans.

### Minor Bacterial Genera Might be Opportunistic or Keystone Players in *S. pistillata*

Based on the presence/absence transformation of abundance, minor genera had significant differences among sampling years, reflecting their fluctuating nature in *S. pistillata* over time. *Ralstonia* and *Propionibacterium* were two acknowledged minor symbiotic genera in corals and intimately associated with dinoflagellate endosymbionts (Ainsworth et al., 2015). However, in our study, *Propionibacterium* was abundantly detected among three locations during sampling, whereas *Ralstonia* had constantly low abundance among sampling sites. Lynch and Neufeld (2015) suggested that prevalent, minor taxa were just conditionally minor in abundance, and would opportunistically grow with abundance under optimal conditions. This accounted for the discrepant abundance of *Propionibacterium* in the current versus previous studies. In contrast, consistent detection of *Ralstonia*’s low abundance described its potential of being a keystone taxon permanently holding rare abundance in coral (Giovannoni and Stingl, 2005; Lynch and Neufeld, 2015), although the functional role of *Ralstonia* in *S. pistillata* remains unclarified.

### Various Functional Roles Are Likely Mediated by Type I Genera in *S. pistillata*

Many bacterial taxa were reported as abundant members in coral (Littman et al., 2009; Liu et al., 2012; McKew et al., 2012; Morrow et al., 2012; Carlos et al., 2013), and mostly on *Spongiobacter* and *Endozoicomonas* (Lee et al., 2012; Morrow et al., 2012; Bayer et al., 2013; Carlos et al., 2013; Lema et al., 2014). These abundantly, frequently detected bacteria seemed adapted to live in coral tissues and acting as coral nutrition, pathogens, probiont, or purely commensal bacteria in the coral holobiont (Klaus et al., 2005). In the present study, we categorized *S. pistillata-*associated bacteria into six resident types, based on their abundance and detection frequency, with *Acinetobacter* (in type I) being the prevalent genera. *Acinetobacter* had been detected in stony corals from various regions (Littman et al., 2009; McKew et al., 2012; Morrow et al., 2012; Carlos et al., 2013; Li et al., 2014), and potentially played either beneficial or detrimental roles in corals. For example, Shnit-Orland and Kushmaro (2009) considered *Acinetobacter* sp. as a first-line defender that assisted the coral holobiont against pathogens resistant to multiple antibiotics. However, in a study of Dark Spot Syndrome in coral *Stephanocoenia intersepta, Acinetobacter* was regarded as a potential pathogen (Sweet et al., 2013). Given the common presence in stony corals, the uncertain role of *Acinetobacter* in *S. pistillata* needs to be clarified.

Among the eight genera in resident type I, four (i.e., *Propionibacterium, Corynebacterium, Arthrobacter*, and *Actinophytocola*) belong to the phylum Actinobacteria, consistent with other reports regarding Actinobacteria as a dominant genus associated with corals. *Propionibacterium* was present in coral *Cirrhipathes lutkeni* (Santiago-Vázquez et al., 2007), *Mussismilia hispida* (de Castro et al., 2010) and *Acropora digitifera* (Nithyanand et al., 2011). Using Actinobacteria-specific primers, Yang et al. (2013) reported 19 actinobacterial genera in two corals, suggesting high diversity of Actinobacteria in both hard and soft corals. Coral-associated actinobacteria reportedly had antimicrobial activity (Sweet et al., 2013; Mahmoud and Kalendar, 2016) attributed to production of bioactive substances (Yang et al., 2013). Therefore, these four type-I actinobacterial genera warrant careful consideration in future studies, considering their broad distribution in corals and antimicrobial properties.

### Metabolic Capability May be Associated with Bacterial Dynamics in *S. pistillata*

In the ocean, heterotrophic bacterial communities (in terms of species composition, spatiotemporal variations, and community structure) are largely affected by the availability, composition, and spatiotemporal distribution of organic substrates. Similarly, variations in coral-associated bacterial community are also controlled, to a great extent, by organic matter secreted by coral, similar to the situation of algae-associated microbial communities (Dang and Lovell, 2016). For example, the genome of *Endozoicomonas* has a wide spectrum of genes related to generic transport for uptake of extracellular organic compounds and carbohydrates (Ding et al., 2016; Neave et al., 2017a). Numbers of transport molecules also implied interactions between *Endozoicomonas* and compounds produced by corals (Neave et al., 2014; Ding et al., 2016). Similarly, *Acinetobacter* was reportedly able to metabolize dimethyl sulfide (Horinouchi et al., 1997) produced by symbiotic algae in corals; furthermore, its precursor (dimethylsulfoniopropionate) has served as a chemoattractant to heterotrophic bacteria in oceans (Seymour et al., 2010). Therefore, availability of extracellular compounds in coral holobiont is likely associated with fluctuations in the abundance of *Endozoicomonas* and *Acinetobacter*.

## Conclusion

This long-term survey revealed dynamics of *S. pistillata*-associated bacterial community (e.g., compositional resilience), with fluctuating patterns of abundant and minor genera stratified by sampling times and geographical locations. Although *Endozoicomonas* and *Acinetobacter* were detected with high abundance and frequency, this long-term observation identified differential inhabiting inclination, defined by *Endozoicomonas* being more acclimated to tropical oceans, whereas *Acinetobacter* was merely confined by climatic zones. Regarding minor genera, despite the lack of functional evidence clarifying their opportunistic or keystone roles in *S. pistillata*, a long-term survey could legitimately distinguish them from somewhat transient colonizers, and also identify microbial candidates for future functional studies. Lastly, abundance-shuffling and species-switching of symbiotic algae improved environmental adaptation of corals (Buddemeier and Fautin, 1993; Fautin and Buddemeier, 2004; Boulotte et al., 2016), although some bacteria had beneficial effects relating to holobiont resilience without abundance fluctuations under short-term heat stress (Ziegler et al., 2017). Therefore, we inferred that large-scale and long-term time series observations are required to characterize fluctuating and steady microbial components in coral holobiont.

## Author Contributions

S-HY substantially analyzed data and wrote the manuscript. C-HT performed bioinformatics analysis, provided detailed methods and results for the analysis, and wrote the manuscript. C-RH performed sampling, DNA extraction, PCR, and data analysis. C-PC acquired samples and performed DNA extraction. KT performed statistical test and participated in the manuscript editing. SL participated in the manuscript writing and editing. P-WC performed sampling, DNA extraction, and PCR. J-HS and CC acquired samples. S-LT planned the experimental design and participated in manuscript editing.

## Conflict of Interest Statement

The authors declare that the research was conducted in the absence of any commercial or financial relationships that could be construed as a potential conflict of interest.

## Footnotes

1 https://github.com/harrelfe/Hmisc
