# Supplementary material for: Long-Term Survey Is Necessary to Reveal Various Shifts of Microbial Composition in Corals
Source: Front Microbiol. 2017 Jun 13;8:1094. doi: 10.3389/fmicb.2017.01094 (PMC5468432; doi:10.3389/fmicb.2017.01094)
Supplement: Supplementary file 1 [file Data_Sheet_1.DOCX]

Supplementary Material

Long term survey is necessary to reveal various shifts of microbial composition in corals

Shan-Hua Yang, Ching-Hung Tseng, Chang-Rung Huang, Chung-Pin Chen, Kshitij Tandon, Sonny T. M. Lee, Pei-Wen Chiang, Jia-Ho Shiu, Chaolun Allen Chen, and Sen-Lin Tang^*^

***Correspondence:** Sen-Lin Tang: sltang@gate.sinica.edu.tw

# Supplementary Tables

## Supplementary Table

**Table S1.** Sampling information and sample identifier

| Sampling location | Sampling time | P*^a^* | S*^b^* | AT*^c^* | ST*^d^* | Coral sample | Seawater sample |
| --- | --- | --- | --- | --- | --- | --- | --- |
| Kenting | 1 | 7.3 | 121.6 | 20.3 | 26.0 | K0802C (n=3) | K0802S |
|  | 2 | 31.5 | 196.8 | 27.0 | 28.0 | K0805C (n=3) | K0805S |
|  | 3 | 653.0 | 204.2 | 27.9 | 28.4 | K0806C (n=3) | K0806S |
|  | 4 | 423.2 | 208.2 | 28.1 | 28.5 | K0808C (n=2) | K0808S |
|  | 5 | 65.5 | 147.2 | 24.5 | 28.0 | K0811C (n=3) | K0811S |
|  | 6 | 9.9 | 152.3 | 23.4 | 25.7 | K0902C (n=3) | K0902S |
|  | 7 | 2.0 | 246.6 | 26.6 | 27.3 | K0905C (n=3) | K0905S |
|  | 8 | 298.6 | 212.8 | 29.1 | 29.6 | K0907C (n=2) | K0907S |
|  | 9 | 3.0 | 170.6 | 24.3 | 26.3 | K0911C (n=2) | K0911S |
|  | 10 | 19.3 | 176.1 | 21.3 | 24.6 | K1001C (n=2) | K1001S |
| Yehliu | 1 | 150.5 | 103.3 | 18.6 | 18.1 | Y0803C (n=3) | Y0803S |
|  | 2 | 145.8 | 154.2 | 27.2 | 25.4 | Y0806C (n=3) | Y0806S |
|  | 3 | 27.1 | 244.1 | 29.4 | 27.3 | Y0808C (n=3) | Y0808S |
|  | 4 | ND*^e^* | ND | ND | ND | ND | ND |
|  | 5 | ND | ND | ND | ND | ND | ND |
|  | 6 | 239.2 | 78.9 | 19.0 | 21.0 | Y0902C (n=3) | Y0902S |
|  | 7 | ND | ND | ND | ND | ND | ND |
|  | 8 | 65.1 | 229.4 | 29.4 | 28.7 | Y0907C (n=3) | Y0907S |
|  | 9 | 611.4 | 62.0 | 23.8 | 24.8 | Y0910C (n=3) | Y0910S |
|  | 10 | 203.2 | 68.6 | 16.9 | 20.5 | Y0912C (n=3) | Y0912S |
| Lyudao | 1 | 22.3 | 102.8 | 21.3 | 24.9 | L0803C (n=3) | L0803S |
|  | 2 | 55 | 163.4 | 26 | 27.4 | L0805C (n=3) | L0805S |
|  | 3 | 159.1 | 241.5 | 28.7 | 28.2 | L0807C (n=3) | L0807S |
|  | 4 | 308.0 | 156.1 | 28.2 | 27.9 | L0809C (n=3) | L0809S |
|  | 5 | 99.9 | 131.1 | 23.4 | 26.0 | L0811C (n=3) | L0811S |
|  | 6 | ND | ND | ND | ND | ND | ND |
|  | 7 | ND | ND | ND | ND | ND | ND |
|  | 8 | 412.1 | 245.7 | 28.9 | 27.9 | L0907C (n=3) | L0907S |
|  | 9 | ND | ND | ND | ND | ND | ND |
|  | 10 | ND | ND | ND | ND | ND | ND |

*^a^*Precipitation (mm)

*^b^*Sunshine duration (h)

*^c^*Air temperature (°C)

*^d^*Seawater temperature (°C)

*^e^*ND, no data

## Supplementary Table

**Table S2.** Diversity indices for bacterial community, as represented in V1–V2 region of 16S rRNA gene.

| Sample | ACE | Chao1 | Cov*^a^* | E*^b^* | OTU | Richness | H*^c^* | Simpson | Singleton OTU | N*^d^* | N for rarefaction |
| --- | --- | --- | --- | --- | --- | --- | --- | --- | --- | --- | --- |
| L0803C | 133.32 | 118.38 | 0.94 | 0.76 | 84.44 | 11.26 | 3.39 | 0.07 | 32 | 11965 | 567 |
| L0805C | 145.14 | 111.12 | 0.94 | 0.69 | 70.99 | 11.26 | 2.94 | 0.11 | 32 | 9341 | 567 |
| L0807C | 216.53 | 162.30 | 0.91 | 0.61 | 97.22 | 18.16 | 2.77 | 0.22 | 51 | 14232 | 567 |
| L0809C | 161.80 | 155.98 | 0.93 | 0.87 | 122.14 | 14.53 | 4.17 | 0.03 | 41 | 3879 | 567 |
| L0811C | 117.40 | 118.85 | 0.95 | 0.86 | 92.40 | 9.81 | 3.87 | 0.03 | 28 | 6149 | 567 |
| L0907C | 183.98 | 163.33 | 0.92 | 0.79 | 113.70 | 17.07 | 3.74 | 0.05 | 48 | 3528 | 567 |
| L0803S | 366.11 | 262.71 | 0.87 | 0.82 | 136.47 | 26.51 | 4.01 | 0.04 | 74 | 808 | 567 |
| L0805S | 628.31 | 405.65 | 0.80 | 0.87 | 188.11 | 40.31 | 4.54 | 0.02 | 112 | 852 | 567 |
| L0807S | 435.67 | 290.82 | 0.85 | 0.86 | 160.65 | 30.51 | 4.39 | 0.02 | 85 | 776 | 567 |
| L0809S | 586.49 | 384.00 | 0.81 | 0.88 | 192.05 | 39.59 | 4.60 | 0.02 | 110 | 1333 | 567 |
| L0811S | 1256.26 | 639.26 | 0.75 | 0.83 | 204.00 | 51.21 | 4.44 | 0.03 | 142 | 567 | 567 |
| L0907S | 448.83 | 368.06 | 0.82 | 0.86 | 181.68 | 36.32 | 4.48 | 0.02 | 101 | 774 | 567 |
| K0802C | 179.76 | 133.98 | 0.93 | 0.67 | 82.96 | 14.16 | 2.96 | 0.14 | 40 | 10418 | 567 |
| K0805C | 162.85 | 136.31 | 0.93 | 0.72 | 91.11 | 13.80 | 3.26 | 0.10 | 39 | 9047 | 567 |
| K0806C | 238.39 | 217.00 | 0.89 | 0.85 | 143.34 | 23.24 | 4.22 | 0.03 | 65 | 11516 | 567 |
| K0808C | 158.04 | 111.18 | 0.94 | 0.56 | 58.68 | 11.26 | 2.27 | 0.20 | 32 | 7323 | 567 |
| K0811C | 112.10 | 109.46 | 0.95 | 0.79 | 89.21 | 9.44 | 3.56 | 0.07 | 27 | 11078 | 567 |
| K0902C | 118.01 | 103.49 | 0.95 | 0.69 | 73.24 | 10.17 | 2.97 | 0.12 | 29 | 15471 | 567 |
| K0905C | 141.24 | 140.05 | 0.94 | 0.88 | 113.39 | 11.62 | 4.16 | 0.02 | 33 | 19557 | 567 |
| K0907C | 87.08 | 86.67 | 0.97 | 0.78 | 69.48 | 6.90 | 3.30 | 0.07 | 20 | 9633 | 567 |
| K0911C | 114.51 | 99.23 | 0.95 | 0.60 | 68.31 | 10.17 | 2.54 | 0.21 | 29 | 4741 | 567 |
| K1001C | 204.90 | 148.97 | 0.92 | 0.58 | 82.58 | 15.98 | 2.56 | 0.22 | 45 | 5439 | 567 |
| K0802S | 3330.53 | 1074.31 | 0.65 | 0.77 | 240.67 | 71.91 | 4.21 | 0.06 | 199 | 2163 | 567 |
| K0805S | 684.35 | 424.44 | 0.78 | 0.87 | 200.80 | 44.67 | 4.59 | 0.02 | 124 | 771 | 567 |
| K0806S | 485.17 | 343.63 | 0.84 | 0.83 | 156.21 | 32.69 | 4.17 | 0.03 | 91 | 2142 | 567 |
| K0808S | 675.61 | 369.06 | 0.82 | 0.71 | 164.39 | 37.77 | 3.61 | 0.13 | 105 | 1124 | 567 |
| K0811S | 1019.46 | 588.98 | 0.70 | 0.93 | 262.49 | 60.65 | 5.16 | 0.01 | 168 | 1561 | 567 |
| K0902S | 490.04 | 328.45 | 0.85 | 0.69 | 141.73 | 31.60 | 3.41 | 0.14 | 88 | 3200 | 567 |
| K0905S | 110.29 | 110.17 | 0.97 | 0.88 | 99.33 | 6.90 | 4.04 | 0.03 | 20 | 2842 | 567 |
| K0907S | 1472.79 | 794.03 | 0.64 | 0.91 | 284.66 | 73.72 | 5.14 | 0.01 | 204 | 1188 | 567 |
| K0911S | 996.52 | 516.15 | 0.78 | 0.79 | 186.90 | 45.40 | 4.15 | 0.06 | 126 | 739 | 567 |
| K1001S | 4257.23 | 1747.52 | 0.40 | 0.96 | 406.15 | 122.39 | 5.74 | 0.00 | 338 | 2162 | 567 |
| Y0803C | 285.99 | 233.49 | 0.87 | 0.68 | 119.33 | 25.79 | 3.25 | 0.10 | 72 | 9051 | 567 |
| Y0806C | 767.21 | 494.59 | 0.75 | 0.87 | 221.57 | 50.48 | 4.71 | 0.02 | 140 | 5073 | 567 |
| Y0808C | 613.50 | 296.87 | 0.86 | 0.72 | 123.80 | 28.33 | 3.44 | 0.08 | 79 | 12698 | 567 |
| Y0902C | 129.61 | 126.31 | 0.95 | 0.83 | 103.47 | 10.90 | 3.85 | 0.04 | 31 | 17788 | 567 |
| Y0907C | 106.50 | 102.45 | 0.95 | 0.75 | 80.79 | 9.08 | 3.31 | 0.08 | 26 | 29819 | 567 |
| Y0910C | 99.22 | 98.84 | 0.97 | 0.81 | 85.98 | 6.90 | 3.62 | 0.05 | 20 | 11201 | 567 |
| Y0912C | 83.76 | 85.12 | 0.98 | 0.89 | 77.52 | 4.00 | 3.86 | 0.03 | 12 | 8785 | 567 |
| Y0803S | 1663.39 | 785.93 | 0.70 | 0.85 | 235.17 | 62.46 | 4.64 | 0.02 | 173 | 2980 | 567 |
| Y0806S | 458.97 | 329.43 | 0.85 | 0.83 | 153.63 | 31.60 | 4.19 | 0.03 | 88 | 1937 | 567 |
| Y0808S | 727.38 | 460.48 | 0.81 | 0.86 | 172.35 | 38.50 | 4.41 | 0.02 | 107 | 4905 | 567 |
| Y0902S | 846.55 | 486.66 | 0.79 | 0.73 | 168.88 | 43.22 | 3.73 | 0.08 | 120 | 10870 | 567 |
| Y0907S | 608.75 | 359.20 | 0.84 | 0.71 | 143.18 | 32.69 | 3.50 | 0.13 | 91 | 7780 | 567 |
| Y0910S | 1112.42 | 622.72 | 0.75 | 0.83 | 202.14 | 50.84 | 4.40 | 0.03 | 141 | 5475 | 567 |
| Y0912S | 850.32 | 503.70 | 0.77 | 0.80 | 196.98 | 47.94 | 4.22 | 0.05 | 133 | 2488 | 567 |

*^a^*Good's coverage = 1 – [(# singleton OTU) / N]

*^b^*Evenness

*^c^*Shannon index

*^d^*Number of total reads

## Supplementary Table

**Table S3.** ANOSIM for abundant and minor bacterial genera in *S. pistillata* stratified by location and time (different sampling years) as factors. Various transformation methods were applied to genera abundance for ANOSIM.

| **Data transformation** | **Factor** | **Abundant genera*^a^*** | **Minor genera*^b^*** |
| --- | --- | --- | --- |
| None (original data) | Location | R=0.240; *p*=0.008 | R=0.043; *p*=0.266 |
|  | Time | R=0.173; *p*=0.028 | R=0.167; *p*=0.024 |
| Square root transformation | Location | R=0.273; *p*=0.002 | R=0.046; *p*=0.257 |
|  | Time | R=0.205; *p*=0.012 | R=0.251; *p*=0.003 |
| Binary transformation (present/absent) | Location | R=0.027; *p*=0.320 | R=0.026; *p*=0.312 |
|  | Time | R=0.075; *p*=0.217 | R=0.227; *p*=0.015 |

*^a^*Bacterial genera ≥1% average relative abundance

*^b^*Bacterial genera <1% average relative abundance

# Supplementary Figures

## Supplementary Figure


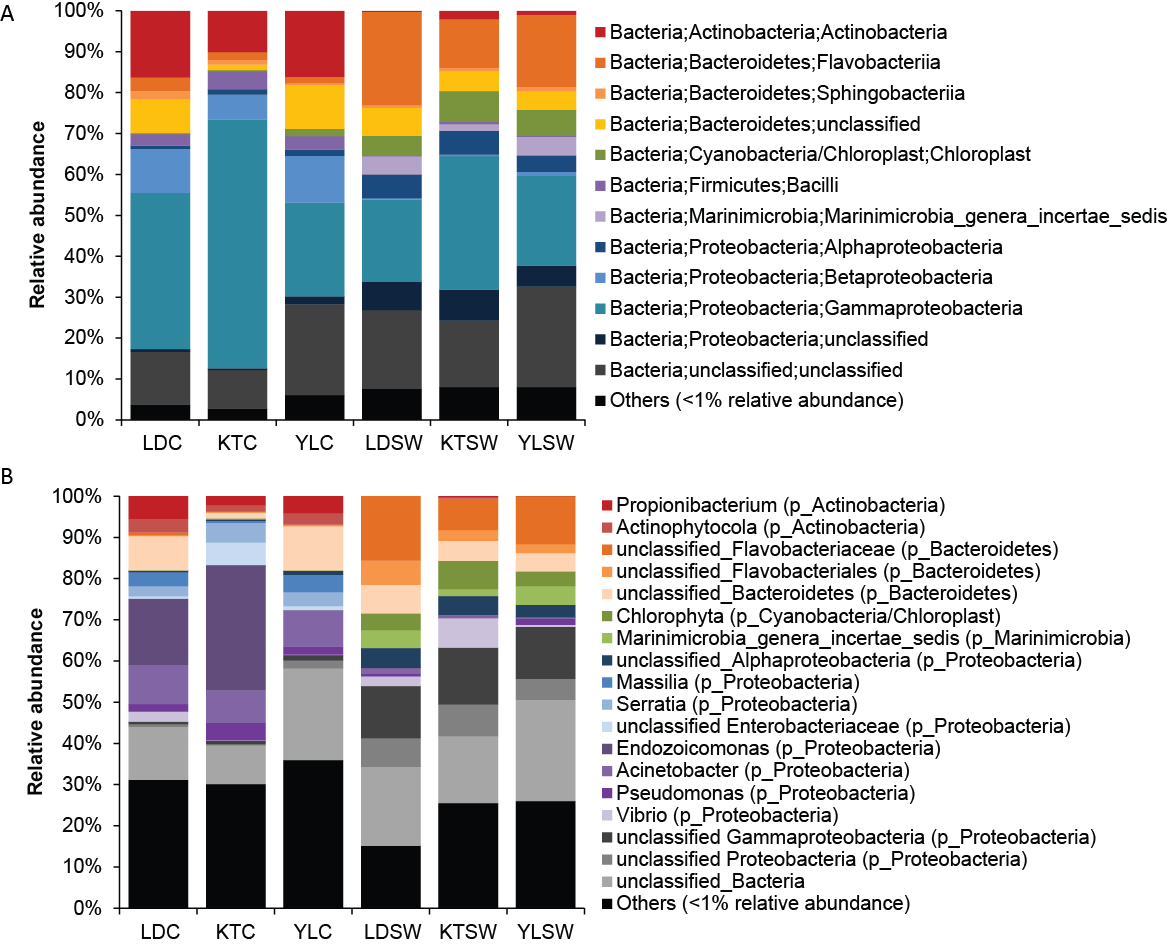


**Figure S1.** Bacterial community composition at (A) class and (B) genus level in *S. pistillata* and seawater. Abundance data were averaged from replicates per sampling origin, including LDC (corals in Lyudao), KTC (corals in Kenting), YLC (corals in Yehliu), LDSW (seawater in Lyudao), KTSW (seawater in Kenting), and YLSW (seawater in Yehliu). The unclassified genus is labeled at the deepest known level prefixed with “unclassified_”, and the phylum name is prefixed with “p_” in the parenthesis. Bacterial classes of <1% relative abundance are collectively shown as “Others”.

## Supplementary Figure


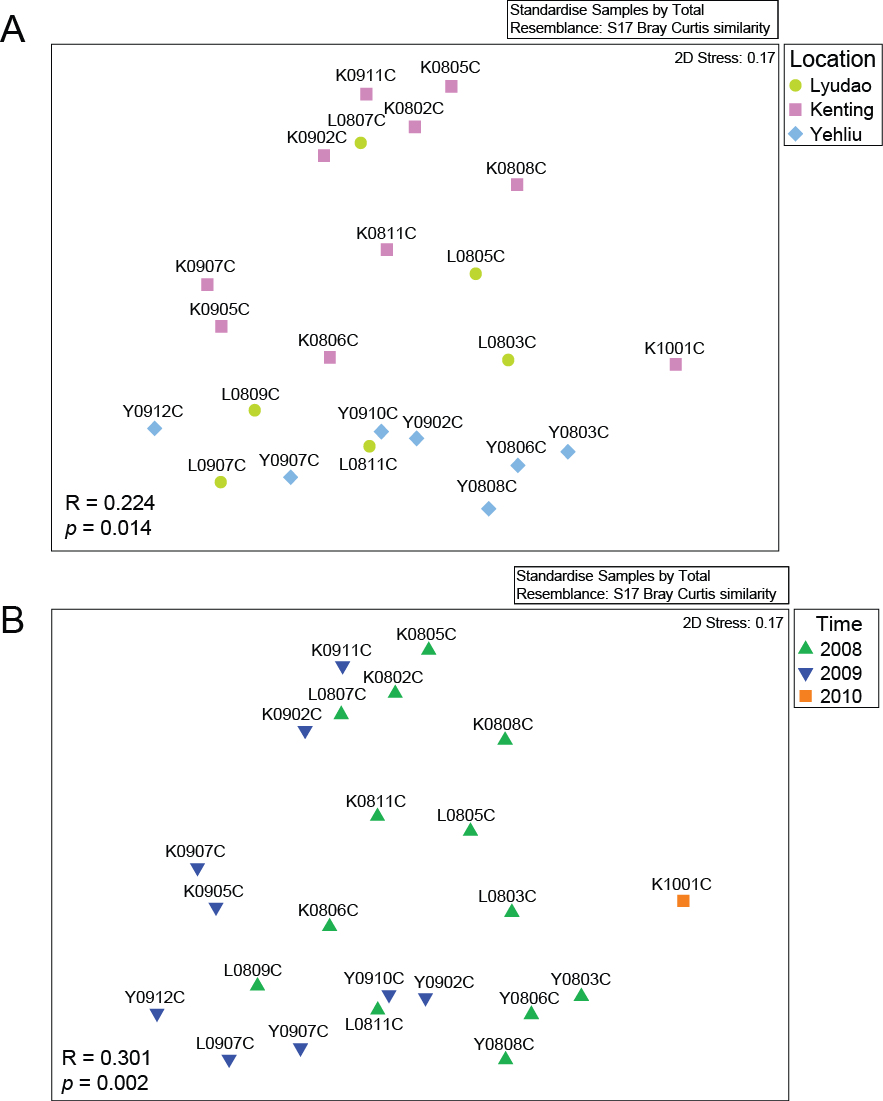


**Figure S2.** nMDS of bacterial communities in *S. pistillata*. (A) Samples were labeled according to locations. (B) Samples were labeled according to sampling years. ANOSIM R value (i.e., the separation strength of given factor on the samples) and *p* value (i.e., significance level) were labeled as inlets in figures.

## Supplementary Figure


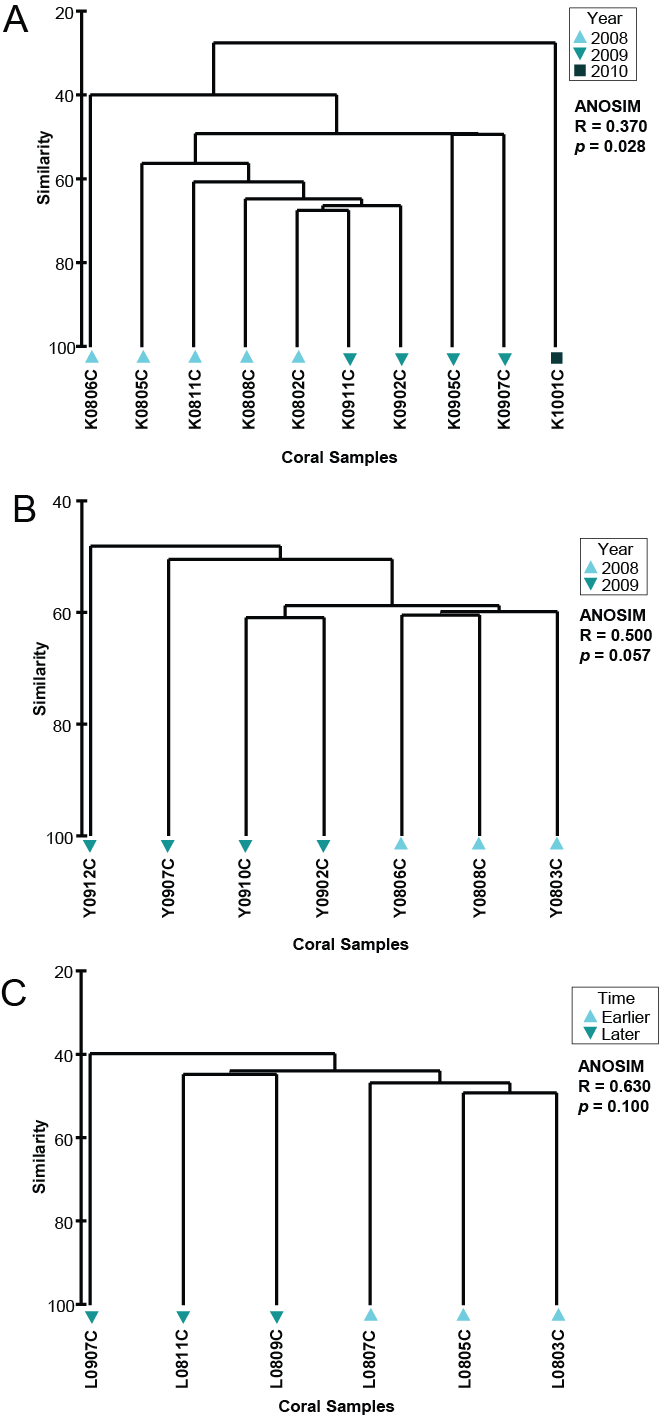


**Figure S3.** Clustering of bacterial communities associated with *S. pistillata* from (A) Kenting, (B) Yehliu, and (C) Lyudao. ANOSIM was performed on each clustering results using sampling year (for Kenting and Yehliu) and sampling time (for Lyudao; earlier, before September 2008; later, after September 2008) as factor. The between-sample similarity (Bray–Curtis distance) was estimated from genus-level relative abundance and used for clustering analysis with single linkage. ANOSIM R value and *p* value were labeled as inlets in figures.

## Supplementary Figure


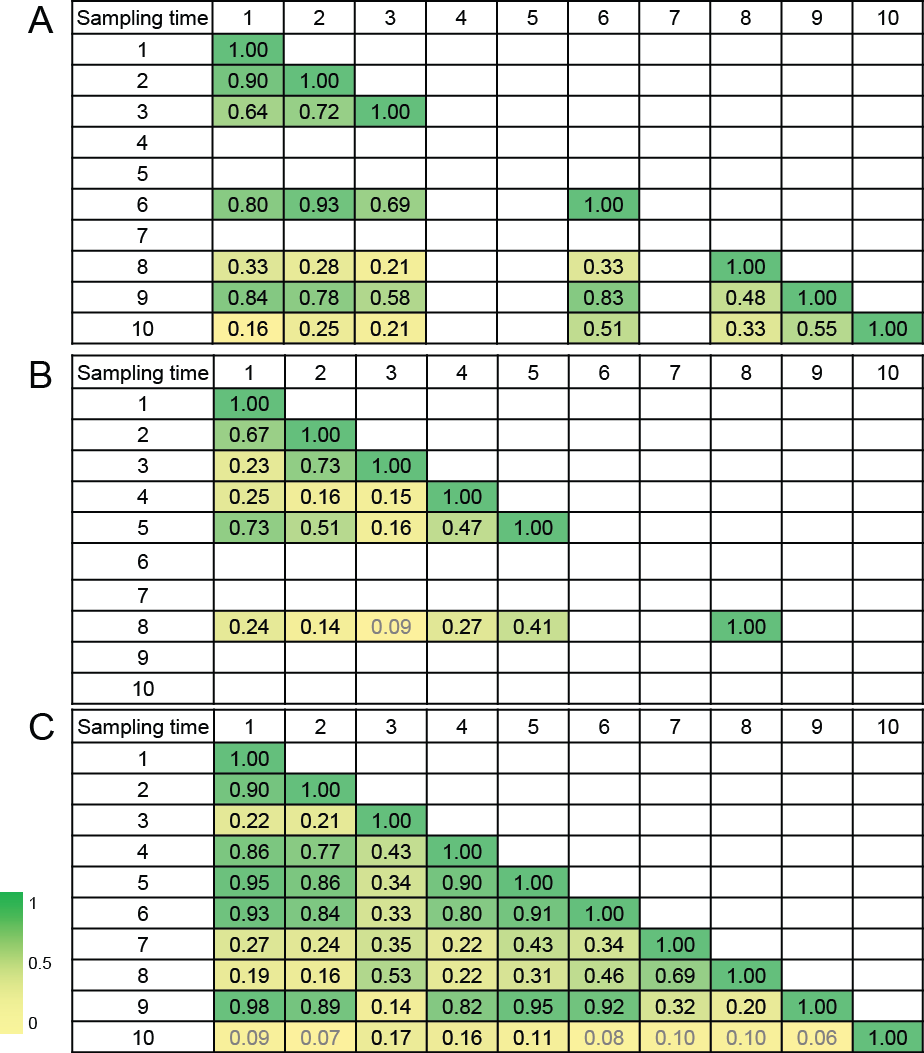


**Figure S4.** Correlation coefficient of bacterial community compositions in *S. pistillata* from (A) Yehliu, (B) Lyudao, and (C) Kenting. Background color in each cell was scaled by Pearson correlation coefficient (*R*) of the given sample pairs in comparison, from 1 (green) for exact correlation to 0 (yellow) for no correlation. Non-significant *R* was shown in grey.

## Supplementary Figure


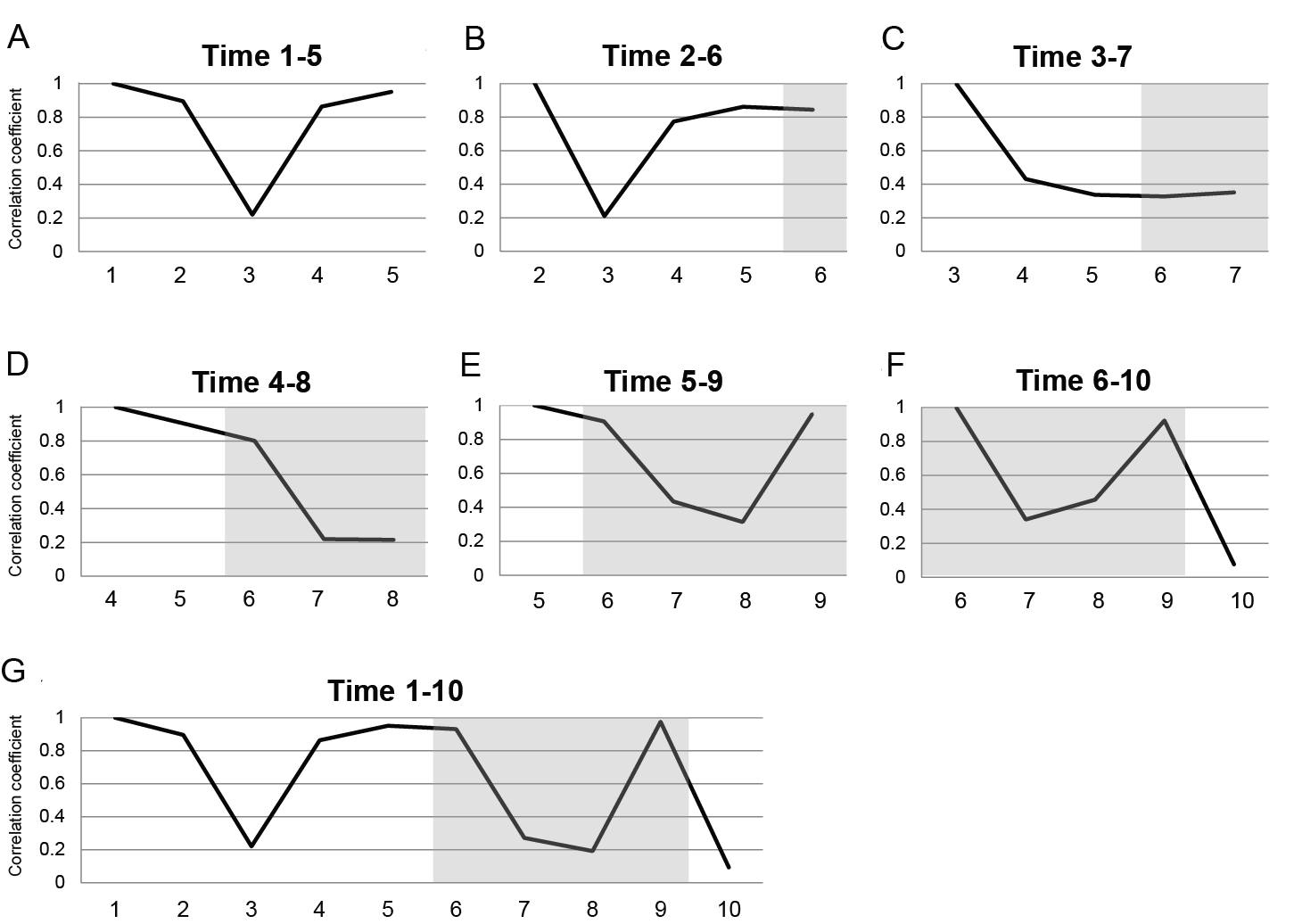


**Figure S5.** Pearson correlation coefficient line chart of bacterial community compositions in *S. pistallata* from Kenting. Each correlation coefficient was calculated between starting and given sampling times. Various short-term intervals (of consecutive five samplings) were shown; (A) from the 1^st^ to 5^th^ sampling, (B) from the 2^nd^ to 6^th^ sampling, (C) from the 3^rd^ to 7^th^ sampling, (D) from the 4^th^ to 8^th^ sampling, (E) from the 5^th^ to 9^th^ sampling, and (F) from the 6^th^ to 10^th^ sampling. The long-term interval was shown in (G), representing the 1^st^ to 10^th^ sampling. The shadow indicated a different year. Peaks indicated high correlation with starting sampling time.
